# Characterisation of macular neovascularisation subtypes in age-related macular degeneration to optimise treatment outcomes

**DOI:** 10.1038/s41433-022-02231-y

**Published:** 2022-09-14

**Authors:** Thibaud Mathis, Frank G. Holz, Sobha Sivaprasad, Young Hee Yoon, Nicole Eter, Lee-Jen Chen, Adrian Koh, Eduardo Cunha de Souza, Giovanni Staurenghi

**Affiliations:** 1 Service d’Ophtalmologie, Centre Hospitalier Universitaire de la Croix-Rousse, Hospices Civils de Lyon, Université Claude Bernard Lyon 1, Lyon, France; 2 UMR5510 MATEIS, CNRS, INSA Lyon, Université Lyon 1, 69100 Villeurbanne, France, Lyon, France; 3 Department of Ophthalmology, University of Bonn, Bonn, Germany; 4 NIHR Moorfields Biomedical Research Centre, Moorfields Eye Hospital NHS Foundation Trust, London, UK; 5 Department of Ophthalmology, Asan Medical Center, University of Ulsan, Seoul, South Korea; 6 Department of Ophthalmology, University of Münster Medical Center, Münster, Germany; 7 Department of Ophthalmology, Mackay Memorial Hospital, Taipei, Taiwan; 8 Camden Medical Centre, Singapore, Singapore; 9 Faculdade de Medicina de Universidade de São Paulo, São Paulo, Brazil; 10 University Eye Clinic, Department of Biomedical and Clinical Science, Luigi Sacco Hospital, University of Milan, Milan, Italy

**Keywords:** Prognostic markers, Retinal diseases

## Abstract

The aim of this review is to identify the common characteristics and prognoses of different subtypes of neovascular age-related macular degeneration (nAMD). We also propose recommendations on how to tailor treatments to the subtype of neovessels to optimise patient outcomes. The authors, selected members of the Vision Academy, met to discuss treatment outcomes in nAMD according to macular neovascularisation (MNV) subtypes, using evidence from a literature search conducted on the PubMed database (cut-off date: March 2019). This review article summarises the recommendations of the Vision Academy on how the characterisation of MNV subtypes can optimise treatment outcomes in nAMD. The identification of MNV subtypes has been facilitated by the advent of multimodal imaging. Findings from fluorescein angiography, indocyanine green angiography and spectral-domain optical coherence tomography collectively help refine and standardise the determination of the MNV subtype. To date, three subtypes have been described in the literature and have specific characteristics, as identified by imaging. Type 1 MNV is associated with better long-term outcomes but usually requires more intense anti-vascular endothelial growth factor dosing. Type 2 MNV typically responds quickly to treatment but is more prone to the development of fibrotic scars, which may be associated with poorer outcomes. Type 3 MNV tends to be highly sensitive to anti-vascular endothelial growth factor treatment but may be associated with a higher incidence of outer retinal atrophy, compared with other subtypes. Accurately assessing the MNV subtype provides information on prognosis and helps to optimise the management of patients with nAMD.

## Abstract (zh)

本综述的目的是确定新生血管性年龄相关性黄斑变性 (nAMD) 不同亚型的特征和预后.并建议如何根据新生血管的亚型调整治疗方案, 以优化患者的预后.作者为视觉学会的选定成员, 根据黄斑区新生血管 (MNV) 的不同亚型, 利用PubMed数据库中检索的文献 (截止日期: 2019年3月),讨论了nAMD的治疗效果.这篇综述文章总结了视觉学会关于不同MNV亚型如何优化nAMD治疗结果的建议.多模式成像促进了MNV亚型的识别.荧光素血管造影术、吲哚菁绿血管造影术和谱域光学相干断层扫描的结果共同完善并标准化了MNV亚型的确定.迄今为止,文献中已经描述了三种亚型,并通过成像确定了它们的具体特征.1型MNV具有更好的长期预后,但通常需要更高剂量的抗血管内皮生长因子.2型MNV通常对治疗反应迅速,但更容易形成纤维化瘢痕,这可能与较差的预后相关.3型MNV往往对抗血管内皮生长因子治疗高度敏感,但与其他亚型相比,可能与更高的视网膜外萎缩发生率相关.准确评估MNV的亚型可提供预后的信息,并有助于优化nAMD患者的管理.

## Introduction

Age-related macular degeneration (AMD) is a leading cause of blindness in elderly people, primarily due to the macular neovascularisation (MNV) and atrophy that can occur during the disease [1, 2]. Since the introduction of anti-vascular endothelial growth factor (VEGF) therapies in 2006, blindness caused by AMD has decreased by 50% in industrialised countries [3]. However, despite the efficacy of anti-VEGF therapies, long-term treatment and follow-up are necessary to maintain visual gains [4]. Although it is well known that more than 85% of patients with neovascular AMD (nAMD) require multiple injections of anti-VEGF therapy after the initial treatment doses [2], a lack of recommendations means patient management in the subsequent treatment period varies widely among ophthalmologists.

Since AMD was first described, many efforts have been made to classify its pathology and the different types of MNV [5–7]. Classification of MNV was originally based on fluorescein angiography (FA), with the neovascular membrane classified as ‘classic’ when new vessels were clearly visible on FA and ‘occult’ when not [8]. Depending on MNV localisation, laser or photodynamic therapy could be recommended treatment options for certain patients [9, 10]. More recent developments in imaging techniques have improved the visualisation of the retina, allowing more precise MNV localisation [11]. Many ophthalmology centres now use multimodal imaging in routine practice and are therefore able to classify MNV subtypes before deciding on a specific treatment regimen. Furthermore, a clearly defined classification of MNV is greatly important as it can help to predict functional and anatomic outcomes after treatment, which can significantly improve patient management.

The aims of this review article are to identify the common characteristics of different MNV subtypes, describe typical nAMD treatment outcomes in each case and propose recommendations on tailoring treatments to the different subtypes.

## Methods

This article is based on a review of the literature and consensus among retinal experts from the Vision Academy. The Vision Academy comprises an international group of retinal physicians who work together to share skills and knowledge and to provide recommendations based on their collective clinical expertise on clinical challenges in areas where there is a lack of conclusive evidence in the literature (www.visionacademy.org).

A literature search was performed on 15 March 2019 using PubMed to identify relevant publications using the following keywords: treatment-naive, anti-VEGF, AMD, type 1, occult, poorly defined, subretinal pigment epithelium, type 2, classic, well defined, subretinal, type 3, retinal angiomatous proliferation, intraretinal, mixed. Manuscripts published in English within 5 years of the date of the literature search (2014–2019) were included. Polypoidal choroidal vasculopathy (PCV) was excluded from this search due to the specific treatment requirements of this neovascular abnormality. A total of 416 publications were initially identified and 75 publications were ultimately reviewed to identify key studies related to MNV subtypes. Additional details on the literature search algorithm are provided in the Supplementary Information.

The objectives of this review are to define and describe MNV subtypes in nAMD and to provide treatment recommendations based on disease characteristics. The recommendations were developed by the authors and subsequently reviewed, commented upon and endorsed by a majority of the Vision Academy membership. Vision Academy members were asked to rate their agreement with the proposed recommendations using the options ‘strongly agree’, ‘agree’, ‘neither agree nor disagree’, ‘disagree’ and ‘strongly disagree’. Responses from more than 50% of members were required for the survey to be valid. Respondents were also asked for the reimbursement status of treatment in their country of practice (i.e., mostly reimbursed or mostly out of pocket) to determine if this may have influenced their responses. Biases were assessed using *χ*^2^. Endorsement was established if 50% or more of respondents indicated that they agreed or strongly agreed. The list of Vision Academy members who have contributed to the recommendations is provided at the end of the article.

## Results

### MNV subtypes can be differentiated by multimodal imaging

The identification of MNV subtypes is facilitated by multimodal imaging. Adding spectral-domain optical coherence tomography (SD-OCT) and indocyanine green angiography (ICGA) to colour fundus photography and FA has been reported to decrease the inter-observer disagreement for neovascular subtype characterisation from 30% to 10% [12]. Although SD-OCT alone is the most reproducible imaging modality for defining neovascular activity, it is less reproducible for defining MNV subtypes, with only moderate intra- and inter-observer agreement [13]. When FA, ICGA and SD-OCT are interpreted together, intra- and inter-observer agreement are almost perfect [13]. FA, ICGA and SD-OCT should therefore be used in conjunction to determine the nAMD subtype.

**Fig. 1:**
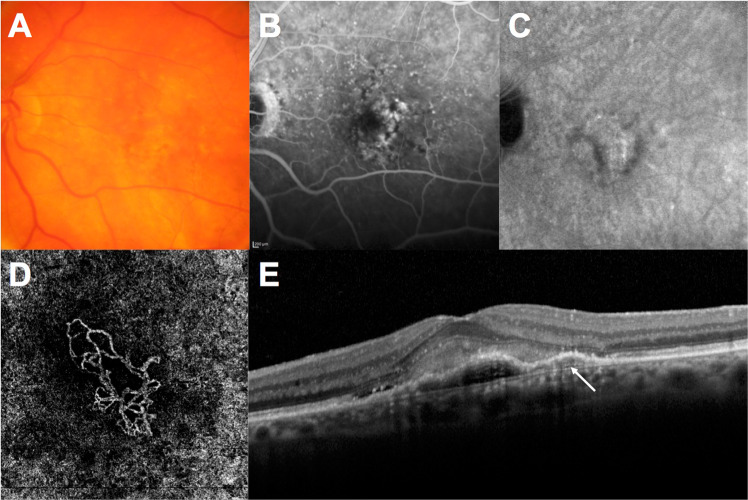
Type 1 macular neovascularisation. **A** Colour fundus photography showing hypo-pigmentary changes of the retinal pigment epithelium; **B** Late phase of fluorescein angiography showing ‘pinpoint’ hyperfluorescence throughout the macular area; **C** Late phase of indocyanine green angiography showing a late hyperfluorescent plaque; **D** Optical coherence tomography angiography demonstrating the neovascular network with large mature vessels; **E** B-scan optical coherence tomography showing pigmentary epithelial detachment associated with a greyish subretinal detachment. Arrow in (**E**) indicates the double layer separating the retinal pigment epithelium from Bruch’s membrane.

Three subtypes of MNV have been described and are characterised through multimodal imaging [11, 14]. Type 1, previously known as ‘occult’ neovascularisation, is characterised by the presence of MNV beneath the retinal pigment epithelial (RPE) layer. FA shows poorly defined late leakage (usually referred to as ‘pinpoints’); ICGA demonstrates a late hyperfluorescent plaque that represents the neovascular network; and SD-OCT shows pigmentary epithelial detachment with no disruption of the RPE layer. A double-layer sign is a common finding of type 1 MNV on SD-OCT and is due to the shallow irregular pigmentary epithelial detachment that splits the upper hyper-reflective band of the RPE from the bottom hyper-reflective band of Bruch’s membrane (Fig. 1). This sign is frequently seen in treatment-naive, quiescent (i.e., non-exudative) type 1 MNV. Another variant of type 1 lesions is PCV occurring on large mature vessels; this can be complicated by recalcitrant oedema or macular haemorrhage [11]. On optical coherence tomography angiography (OCT-A), PCV lesions can demonstrate round hyper-flow structures surrounded by hypo-intense ‘halos’, the latter thought to be due to low flow signals [15]. However, polypoidal lesions have demonstrated variable patterns on OCT-A and are not always detected. Multimodal imaging, especially ICGA, can more clearly detect polyps and may more accurately diagnose this lesion type [16]. Patients with this vascular abnormality often require more frequent anti-VEGF treatment and eventually photodynamic therapy [17].

**Fig. 2:**
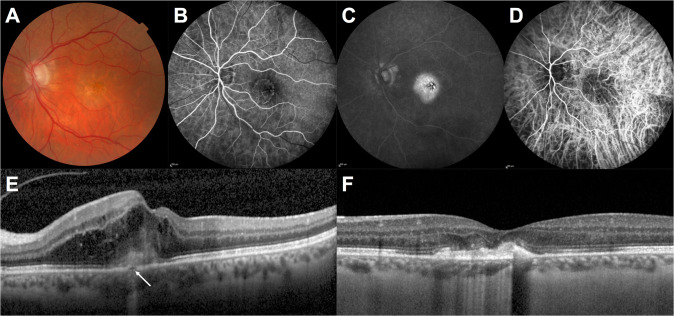
Type 2 macular neovascularisation. **A** Colour fundus photography showing hyper- and hypo-pigmentation of the retinal pigment epithelium; **B** Early phase of fluorescein angiography showing the neovascular membrane; **C** Late phase of fluorescein angiography showing macular leakage of the neovessels; **D** Indocyanine green angiography showing the neovascular network; **E** B-scan optical coherence tomography showing disruption of the retinal pigment epithelium (arrow) by the neovascular complex, which is present above this layer. Intraretinal fluid is present in the macular area; **F** B-scan optical coherence tomography 1 year after the start of disease. A fibrotic scar prevents visual recovery.

Type 2 MNV, previously known as ‘classic’ neovascularisation, is characterised by the presence of MNV of choroidal origin in the neuroretina, having broken through the RPE layer. FA shows a well-defined neovascular membrane, with intense leakage that increases over time. While the contribution of ICGA is less important to the diagnosis of type 2 MNV, it can reveal a sub-RPE part of the neovascular membrane, defining the MNV as a mixed lesion with both type 1 and type 2 components. SD-OCT shows a disruption of the RPE–Bruch’s membrane complex and localisation of the neovessels above the RPE layer [11] (Fig. 2).

**Fig. 3:**
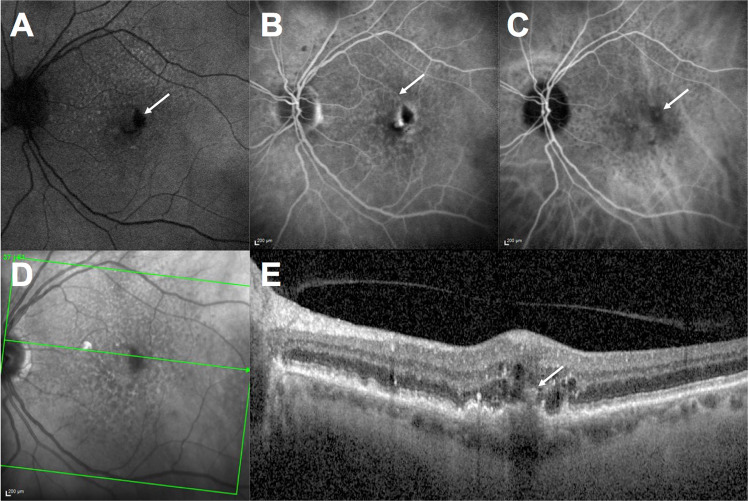
Type 3 macular neovascularisation. **A** Fundus autofluorescence showing macular haemorrhage (arrow) and the presence of reticular pseudodrusen, represented by hypo- and hyperautofluorescence in the superior part of the macular area; **B** Fluorescein angiography showing parafoveal leakage located at the end of a retinal vessel (arrow); **C** Indocyanine green angiography showing mild hyperfluorescence (arrow) at the distal part of the retinal vessel; **D** Infra-red imaging showing a hyper- and hyporeflectivity pattern on the macular area, characteristic of retinal pseudodrusen; **E** B-scan optical coherence tomography showing a pigment epithelial detachment with retinal pigment epithelial disruption and a hyper-reflective lesion (arrow) in the outer retinal layer. Moderate intraretinal fluid is also present.

Type 3 MNV, also known as retinal angiomatous proliferation (RAP), is characterised by anomalous vascular complexes originating in the neuroretinal layers [11]. FA shows early focal leakage close to retinal vessels, ICGA demonstrates a late hyperfluorescent hot spot, and SD-OCT contributes significantly to the diagnosis of and provides information on the stage of disease [11, 18]. Stage 1 involves an intraretinal hyper-reflective lesion in front of a pigmentary epithelial detachment, associated with mild cystoid macular oedema without outer retinal alterations. Stage 2 involves an outer retinal alteration with RPE disruption and an increase in the hyper-reflective lesion, in addition to the intraretinal oedema. Stage 3 is defined by intraretinal hyper-reflective lesions that extend through the RPE to vascularise the pigmentary epithelial detachment [18]. A ‘kissing sign’ between the inner retinal layers and the RPE is frequently present at this disease stage and subretinal fluid can occur (Fig. 3).

The role of OCT-A in distinguishing the different MNV subtypes has yet to be clarified [19–21]. OCT-A has demonstrated high diagnostic value in detecting choroidal neovascularisation in nAMD [22], but it is not clear how this technology can help determine MNV subtypes, and the designs and sample sizes of almost all studies using OCT-A have not allowed for the characterisation of neovessels. Although OCT-A can highlight intraretinal flow in RAP [20], it is currently impossible to determine the precise localisation of neovessels with respect to the RPE layer. However, it has been shown that type 1 lesions exhibit mature vessels [21] and a larger area of neovascularisation than type 2 lesions [19]. Interestingly, several papers have reported quiescent or non-exudative type 1 MNV detected by OCT-A in fellow eyes in nAMD or in eyes with geographic atrophy (GA) [23, 24]. These type 1 lesions have been associated with reduced localised progression of atrophy, which may have clinical implications for their management [25].

### Natural history of choroidal neovascularisation differs according to neovascular subtype

The various subtypes of nAMD are known to be associated with variable visual outcomes. Occult (type 1) MNV may have better outcomes if left untreated, and the lesion can be stable for months or years [26, 27]. In one study, 53.4–64.5% of eyes with a type 1 lesion lost three lines of visual acuity (VA) at 1 year, but VA remained stable in up to 30% of eyes [26]. Another study reported a median VA loss of 2.5 lines at 1 year [27] and, in the MARINA study, VA loss at 1 and 2 years was 10.4 and 14.9 letters, respectively [28]. In contrast, classic (type 2) MNV is associated with poor outcomes and the development of more fibrotic scars if undertreated or untreated [9, 10]. Approximately 60% of untreated eyes lose three lines or more at 1 year [10] and the mean VA loss at 2 years is about four lines [9]. Finally, type 3 MNV is associated with the worst outcomes if left untreated. Viola et al. [29] reported that 69% of eyes with RAP (type 3) MNV had VA of 20/200 or less, 36% of patients were legally blind at 1 year, and the mean decrease in VA was around six lines at 1 year. In a meta-analysis of untreated control eyes of various MNV subtypes in randomised controlled trials, baseline VA, rather than angiographic classification, appeared to be the major determinant of the variation in VA over time [8].

A drawback of these studies is that they used only FA to classify eyes as having occult MNV, classic MNV or RAP. As such, some patients might have been diagnosed incorrectly (e.g., some patients with type 3 MNV might have been classified as having minimally classic [type 2] MNV). Indeed, in both interventional clinical trials and observational studies, the reported rates of each neovascular subtype vary. For example, in a Brazilian prospective epidemiology study, 62.6% of eyes with nAMD had type 1 or 2 MNV, 12.8% had type 3 MNV and 24.5% had PCV [30]. In the Comparison of Age-Related Macular Degeneration Treatments Trial (CATT), 42.0–48.3% of lesions were type 1, 19.2–23.7% were type 2, 11.4–14.3% were mixed and 9.6–11.7% were type 3 [31]. In an observational, retrospective, real-life study, multimodal imaging showed that 39.9% of lesions were type 1, 9.0% were type 2, 16.9% were mixed and 34.2% were type 3 [14]. This variation indicates that some subtypes are likely to be misdiagnosed or excluded in some studies, thus not reflecting routine practice. Moreover, lesions can progress over time from one choroidal neovascularisation subtype to another. Although there are some case series in the literature reporting the evolution of type 2 to type 1 MNV [32], a study from 2005 reported that approximately a quarter of eyes progressed from ‘occult’ type 1 MNV to neovascularisation with a ‘classic’ type 2 component over a review period of 6–12 months [33].

For these reasons, MNV subtypes need to be carefully characterised so that study data can be correctly interpreted.

### Treatment outcomes may depend on the neovascular subtype

Precise characterisation of MNV is important, as treatment outcomes may depend on the subtype. Compared with other subtypes, eyes with type 1 MNV have been reported to be more likely to maintain vision over time, despite requiring more frequent anti-VEGF injections in a treat-and-extend (T&E) regimen (approximately nine injections per year in a 5-year follow-up) [34, 35]. Moreover, type 1 lesions had 6.7 times less risk of developing GA than eyes with other lesion subtypes [36]. Studies have shown that the progression of GA is reduced in treatment-naive, quiescent, as well as formerly exudative, type 1 MNV [25, 36]. The question of whether or not to treat quiescent MNV is not well studied in the literature due to a lack of long-term outcomes. However, tolerating some subretinal fluid (which is a major finding in patients with type 1 MNV) in a T&E regimen has been reported to achieve similar results with fewer injections compared with a more restrictive protocol [37].

Type 2 MNV is associated with more fibrotic scarring than other MNV types [9, 10], which is a major risk factor for poor visual outcomes after treatment [38]. In a *post hoc* analysis of the CATT study, type 2 lesions had a 4.5-fold higher risk of developing a fibrotic scar compared with type 1 lesions [39]. In addition, a separate study reported that eyes with subretinal hyper-reflective material at baseline that led to subretinal fibrosis were more often diagnosed with type 2 lesions [40]. However, type 2 MNV typically responds faster to anti-VEGF therapy than type 1 lesions, as the time between diagnosis and inactivation of the lesion is shorter for this subtype, regardless of injection frequency. The small lesion size and localisation of the MNV complex above the RPE cell monolayer could explain this faster response to treatment [41], and patients generally need fewer injections than those with other MNV subtypes [35].

Type 3 lesions are more prone to responding to anti-VEGF therapy, with the small size of the neovascularisation and its intraretinal localisation likely to lead to better exposure to treatment. In a recent study, VA and VA gains at 1 and 2 years were better in type 3 MNV compared with other nAMD subtypes [42]. In the CATT study, although the mean improvement in VA from baseline was greater for RAP lesions at 1 year, it was similar to other subtypes at 2 years. The more frequent extrafoveal position of the lesion accounted for the relatively good short-term visual outcomes [43]. However, long-term studies have reported higher rates of GA in eyes with type 3 neovascularisation, with up to 86% of patients developing atrophy during follow-up [44–46]. In the CATT study, type 3 MNV was found to be a significant predictive factor for developing atrophy at 2 and 5 years [47, 48]. This subtype is associated with a thin choroid and frequent retinal pseudodrusen, which could explain the high rate of GA [43]. Type 3 MNV was more often inactive at 2 years, although the median number of injections was similar compared with the group with types 1 and 2 MNV [42]. However, if the lesion was treated at an earlier stage, the total number of injections needed at 1 year decreased [49]. Moreover, visual outcomes were worse in stage 3 than in stage 2, and adverse events that may lead to abrupt visual deterioration developed in stage 3 only [50].

### The risk of relapse varies between MNV subtypes

The risk of relapse and the involvement of the fellow eye vary between MNV subtypes. Type 3 lesions have a higher risk of being associated with pathology in the fellow eye, so more aggressive follow-up should be implemented in the presence of this lesion type [51, 52]. In a study by Bochicchio et al. [51], 38% of patients with newly diagnosed RAP lesions suffered from MNV in the fellow eye at 3 years, compared with 11% of patients with type 1 and 6% with type 2 MNV. In addition, half of the fellow eyes with type 3 MNV had developed neovascularisation by 3.5 years, compared with 5.3 years for other subtypes [51].

It is not clear from the literature whether the rate of recurrence is higher in eyes with type 3 lesions, due to the variation in published results. In the PrONTO study, RAP lesions required more injections than the other MNV subtypes, indicating a higher rate of recurrence [53]. Conversely, in the CATT study, RAP lesions required fewer injections compared with the other subtypes [43]. In real-life practice, recurrence of type 3 MNV occurs in around 80% of eyes within a mean of 4–6 months after the initial treatment doses [54]. However, treating early-stage RAP appears to result in fewer recurrences and better visual outcomes compared with treating later stages. In a study by Park and Roh [49], a majority of patients with stage 1 RAP did not experience any relapse during the first year after an initial treatment dose of three anti-VEGF injections.

## Discussion and recommendations

**Fig. 4:**
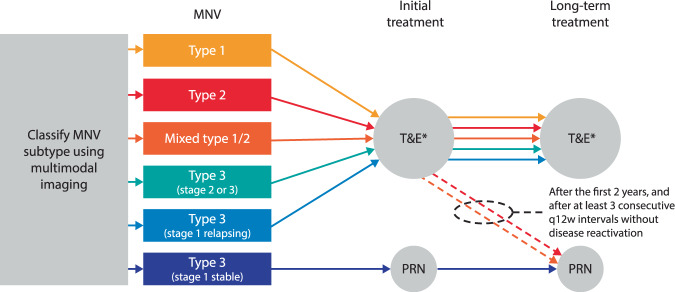
Decision tree on how to tailor the treatment regimen according to MNV subtype. *Fixed should not be the treatment regimen of choice but can be used in some instances where T&E is not feasible due to resource or organisational constraints. MNV macular neovascularisation, PRN *pro re nata* (as needed), q12w once every 12 weeks, T&E treat-and-extend.

To optimise functional outcomes in patients with nAMD, the treatment regimen should be individualised for each patient, according to the type of MNV. In all cases, treatment should be initiated promptly, as early as possible. Characterising the neovascular subtype can provide information on the expected prognosis, response to treatment and involvement of the fellow eye; therefore, multimodal imaging, ideally including FA, ICGA and OCT (although this may not always be feasible), should be used to accurately classify the lesion as type 1, 2 or 3 MNV. While OCT-A can help in detecting neovessels when they are not clearly visible on classic examinations, it cannot characterise the MNV subtype on its own. Correct multimodal assessment of the MNV subtype can help guide practitioner decisions on choosing an adapted treatment regimen. The following recommendations for treatment according to MNV subtype have been developed and endorsed by the Vision Academy members (Fig. 4; Table 1).

**Table 1:** Vision Academy treatment recommendations for nAMD according to MNV subtype.

MNV subtype	Treatment recommendations
All subtypes	• Multimodal imaging, ideally including FA, ICGA and OCT, should be used to accurately classify the lesion wherever possible
Type 1 (occult neovascularisation)	• An individualised T&E regimen • Extended treatment and observation periods are required
Type 2 (classic neovascularisation)	• For both purely type 2 lesions and mixed lesions with both type 1 and 2 components, an intensive T&E regimen in the first 2 years, extending beyond q12w if possible (after at least three consecutive q12w intervals without disease reactivation) • After the first 2 years of an intensive T&E regimen, purely type 2 lesions can be managed with careful PRN treatment with frequent monitoring • After the first 2 years of an intensive T&E regimen, mixed lesions can be managed on a case-by-case basis with T&E or careful and frequently monitored PRN treatment
Type 3 (retinal angiomatous proliferation)	• Stage 1 lesions that have reached stability after three treatment initiation doses can be treated with a strict (monthly) PRN regimen with contralateral eye checks • Patients with non-stable or relapsing stage 1 lesions may be switched to a proactive regimen (T&E or fixed) • Stage 2/3 lesions should be treated with a proactive regimen

*FA* fluorescein angiography, *ICGA* indocyanine green angiography, *MNV* macular neovascularisation, *nAMD* neovascular age-related macular degeneration, *OCT* optical coherence tomography, *PRN* *pro re nata* (as needed), *q12w* once every 12 weeks, *T&E* treat-and-extend.

Type 1 MNV often requires more anti-VEGF injections than other types of MNV, as large mature vessels may be present in the neovascular complex and may produce recalcitrant or persistent subretinal exudation. However, long-term outcomes are typically better. As such, an individualised regimen such as T&E should be proposed as a priority to reduce patient burden. Alternatively, a fixed-dose regimen could be proposed depending on the observed time to recurrence, where T&E is not feasible due to resource or organisational constraints. Long-term treatment and follow-up are necessary, as the large vessels forming type 1 MNV are prone to developing PCV and its related complications such as subretinal and choroidal haemorrhage.

Type 2 MNV usually responds quickly to anti-VEGF therapy but is prone to the development of fibrotic scars. Type 2 lesions may be treated using an intense T&E regimen in the first 2 years, extending beyond once every 12 weeks (q12w) if possible. After the first 2 years, purely type 2 lesions can be managed with careful and frequently monitored *pro re nata* (as needed) treatment (after at least three consecutive q12w intervals without disease reactivation). Mixed lesions, with both type 1 and type 2 components, can be monitored using an intense T&E regimen in the first 2 years. After the first 2 years, mixed lesions can be managed on a case-by-case basis with T&E or careful and frequently monitored *pro re nata* treatment (after at least three consecutive q12w intervals without disease reactivation).

Type 3 lesions tend to be very sensitive to anti-VEGF therapy, and treating lesions early leads to better visual outcomes with fewer recurrences and injections. However, the incidence of GA appears to be higher than in other MNV subtypes, and the fellow eye frequently develops neovascular complications. Further studies are needed to determine whether eyes with type 3 lesions at high risk of GA might be safely managed with a *pro re nata* regimen. At present, patients with type 3 stage 1 lesions, having reached stability after three initial treatment doses, can be kept on a strict (monthly) *pro re nata* regimen with contralateral eye checks. Patients with non-stable or relapsing stage 1 lesions should be switched to a proactive regimen (T&E or fixed). Patients with type 3 stage 2/3 lesions should be treated with a proactive regimen.

In this review, we have excluded PCV from the literature search as this neovascular abnormality generally requires more intensive anti-VEGF treatment than other MNV types, and often needs additional treatment modalities such as photodynamic therapy. Due to the evolving treatment paradigms for PCV, this vascular abnormality should be considered, especially when the initial imaging is not typical of other MNV subtypes or when treatment outcomes are not as expected.

## Conclusion

Correct assessment of the MNV subtype provides information on a patient’s prognosis and helps to determine the preferred treatment regimen. Additional biomarkers, perhaps as found on OCT-A, are needed to better optimise treatment outcomes.

Supplementary Information is available on Eye’s website.

## Supplementary information

Supplemental methods
