# Supplementary material for: Characterisation of macular neovascularisation subtypes in age-related macular degeneration to optimise treatment outcomes
Source: Eye (Lond). 2022 Sep 14;37(9):1758–65. doi: 10.1038/s41433-022-02231-y (PMC10275926; doi:10.1038/s41433-022-02231-y)
Supplement: Supplementary file 1 — Supplemental methods [file 41433_2022_2231_MOESM1_ESM.docx]

**Supplementary Information**

Additional details describing the literature search algorithm used to identify relevant publications for this review article.

**Methods**

The keywords listed in the Methods section were divided into four groups. Group 1 terms were treatment-related and included ‘anti-VEGF’ OR ‘anti-vascular endothelial growth factor’ OR ‘anti-angiogenic agent’ OR ‘bevacizumab’ OR ‘ranibizumab’ OR ‘aflibercept’ OR ‘Avastin’ OR ‘Lucentis’ OR ‘Eylea’. Group 2 terms were condition-related and included ‘age-related macular degeneration’ OR ‘AMD’ OR ‘ARMD’. Group 3 terms were subject-related and included ‘subtype’ OR ‘type 1’ OR ‘occult’ OR ‘poorly defined’ OR ‘subretinal pigment epithelium’ OR ‘type 2’ OR ‘classic’ OR ‘well defined’ OR ‘subretinal’ OR ‘type 3’ OR ‘retinal angiomatous proliferation’ OR ‘intraretinal’ OR ‘mixed’. Group 4 terms were patient-related and included ‘treatment-naïve’ OR ‘treatment naïve’ OR ‘naïve’.

The literature search was performed using the following algorithm: search for any terms in group 1 (treatment-related) in combination with each of the terms in group 2 (condition-related), group 3 (subject-related) and group 4 (patient-related).
